# Supplementary material for: Effectiveness and Safety of Ayurvedic Medicines in Type 2 Diabetes Mellitus Management: A Systematic Review and Meta-Analysis
Source: Front Pharmacol. 2022 Jun 8;13:821810. doi: 10.3389/fphar.2022.821810 (PMC9213670; doi:10.3389/fphar.2022.821810)
Supplement: Supplementary file 1 [file DataSheet1.zip › Supplementary Material/Forest and Funnel Plots/Zingiber officinale/HbA1c.pdf]

Zingiber officinale - HbA1c

Study	Intervention N	Intervention Mean	Intervention SD	Comparator N	Comparator Mean	Comparator SD	Mean Diff. with 95% CI	Weight (%)
Mahluji 2013	28	6.70	1.40	30	6.80	1.50	-0.10 [-0.85, 0.65]	10.20
Arablou 2014a, Arablou 2014b	33	7.30	1.30	30	8.60	2.20	-1.30 [-2.18, -0.42]	8.59
Azimi 2014, Azimi 2016	41	7.90	0.43	39	7.51	0.62	0.39 [0.16, 0.62]	17.86
Mozaffari-Khosravi 2014, Talaei 2017, Talaei 2018	40	7.70	1.70	41	8.20	1.90	-0.50 [-1.29, 0.29]	9.72
Shidfar 2015	22	6.60	1.26	23	7.30	1.32	-0.70 [-1.45, 0.05]	10.11
Arzati 2017, Zarezadeh 2018	23	6.92	1.93	22	7.72	2.08	-0.80 [-1.97, 0.37]	6.03
Mohammadi 2017, Mohammadi 2019	22	8.21	0.50	22	8.24	0.53	-0.03 [-0.33, 0.27]	16.89
Carvalho 2020	47	8.14	1.81	56	8.29	1.86	-0.15 [-0.86, 0.56]	10.67
Gholinezhad 2020	21	7.84	1.48	21	8.18	1.02	-0.34 [-1.11, 0.43]	9.93
**Overall**							-0.27 [-0.62, 0.07]	

Heterogeneity: $\tau^2 = 0.16$, $I^2 = 68.58\%$, $H^2 = 3.18$

Test of $\theta_i = \theta_j$: Q(8) = 26.56, p = 0.00

Test of $\theta = 0$: z = -1.55, p = 0.12

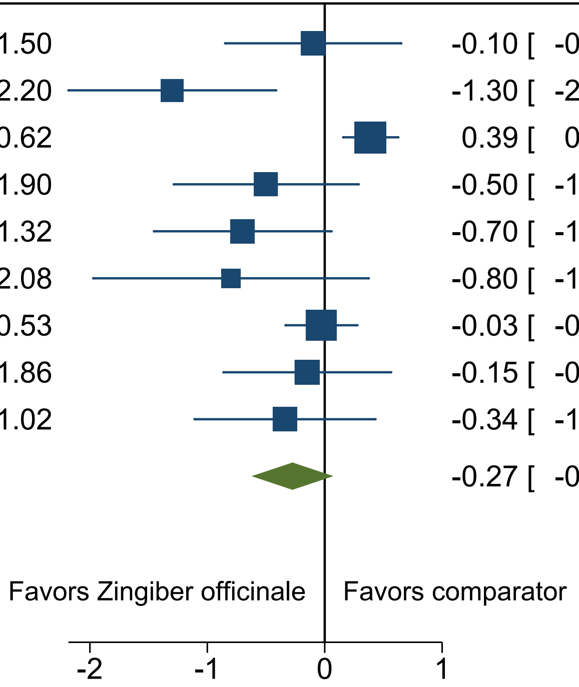

Random-effects REML model
